# Widefield Optical Coherence Tomography in Pediatric Retina: A Case Series of Intraoperative Applications Using a Prototype Handheld Device

**DOI:** 10.3389/fmed.2022.860371

**Published:** 2022-07-04

**Authors:** Thanh-Tin P. Nguyen, Shuibin Ni, Guangru Liang, Shanjida Khan, Xiang Wei, Alison Skalet, Susan Ostmo, Michael F. Chiang, Yali Jia, David Huang, Yifan Jian, J. Peter Campbell

**Affiliations:** ^1^Casey Eye Institute, Oregon Health and Science University, Portland, OR, United States; ^2^Department of Biomedical Engineering, Oregon Health and Science University, Portland, OR, United States; ^3^Knight Cancer Institute, Oregon Health and Science University, Portland, OR, United States; ^4^Department of Radiation Medicine, Oregon Health and Science University, Portland, OR, United States; ^5^Department of Dermatology, Oregon Health and Science University, Portland, OR, United States; ^6^National Eye Institute, National Institutes of Health, Bethesda, MD, United States

**Keywords:** retina, pediatric retina, optical coherence tomography, handheld optical coherence tomography, optical coherence tomography with angiography

## Abstract

Optical coherence tomography (OCT) has changed the standard of care for diagnosis and management of macular diseases in adults. Current commercially available OCT systems, including handheld OCT for pediatric use, have a relatively narrow field of view (FOV), which has limited the potential application of OCT to retinal diseases with primarily peripheral pathology, including many of the most common pediatric retinal conditions. More broadly, diagnosis of all types of retinal detachment (exudative, tractional, and rhegmatogenous) may be improved with OCT-based assessment of retinal breaks, identification of proliferative vitreoretinopathy (PVR) membranes, and the pattern of subretinal fluid. Intraocular tumors both benign and malignant often occur outside of the central macula and may be associated with exudation, subretinal and intraretinal fluid, and vitreoretinal traction. The development of wider field OCT systems thus has the potential to improve the diagnosis and management of myriad diseases in both adult and pediatric retina. In this paper, we present a case series of pediatric patients with complex vitreoretinal pathology undergoing examinations under anesthesia (EUA) using a portable widefield (WF) swept-source (SS)-OCT device.

## Introduction

Optical coherence tomography (OCT) is an essential diagnostic tool in the management of retinal disease. There are trade-offs in the acquisition of OCT images between speed of acquisition, field of view (FOV), and image resolution and quality. Over the last two decades, despite significant advances in imaging speed and the transition from time-domain to spectral domain (SD)-OCT, the vast majority of OCT applications are for macular diseases in adults. OCT has proven ability to detect subclinical disease, often resulting in new disease classifications and earlier treatment, facilitate objective assessment of macular thickness and pathologic fluid, and improve visualization of the vitreoretinal interface. As a result, it is not possible to provide the standard of care for many adult retinal diseases without OCT.

These same advances in clinical diagnosis and management would likely benefit pediatric retina patients. In retinopathy of prematurity (ROP), the most common pediatric retinal disease, OCT has revealed the normal spectrum of macular development in prematurely born infants (1, 2), identified the presence of intraretinal fluid (3, 4), and demonstrated the ability to objectively assess changes at the vitreoretinal interface (5). However, early work has been limited by the specifications of commercially available devices. Over the past few years, a number of groups have explored the advantages of arm-mounted SD-OCT (6) and prototype swept-source (SS)-OCT in pediatric retinal diseases (7–10). With the versatility of a handheld probe and faster image acquisition times, SS-OCT has improved the ease of imaging in both awake and sedated children.

We have developed a handheld SS-OCT device with two imaging configurations, one with a 55° FOV and higher resolution for OCTA imaging (11), and one with a 105° FOV for OCT structural imaging only (12). Our 55° FOV system generates OCTA volumes concurrently with OCT, and both imaging configurations allow for real time *en face* visualization to allow the physician to position the probe optimally for image acquisition. The 105° FOV system has potential to provide objective diagnosis in pediatric retinal diseases with predominantly extramacular pathology, like ROP, and contribute to new insight in these disease processes. We recently described our experience using these devices for ROP screening in the neonatal intensive care unit (NICU) in awake infants (13, 14). Here, we present a review of the potential clinical benefits and applications of widefield (WF) and ultra-widefield (UWF) handheld OCT in pediatric retina patients undergoing examinations under anesthesia (EUA) for a variety of conditions.

## Materials and Methods

**FIGURE 1:**
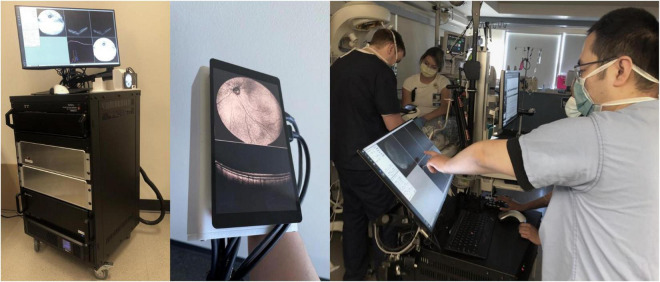
Handheld, SS-OCT device. From left: portable prototype device, imaging probe with real-time *en face* display, and process of obtaining OCT volumes of an infant in the NICU.

This study was approved by the Institutional Review Board (IRB) at Oregon Health and Science University (OHSU) and adheres to all tenets of the Declaration of Helsinki. Consent for imaging was obtained from parents. Pupils were pharmacologically dilated per routine clinical care. Infants were imaged in the operating room (OR) after the induction of general anesthesia and placement of an eyelid speculum with a 400-kHz portable handheld SS-OCT system, shown in Figure 1, using a modular lens system providing up to a 105° FOV. A display screen on the probe provides real-time *en face* visualization of the retina and allows for efficient positioning of the probe (15–17). The probe was operated by the examining ophthalmologist, whilst another operator controlled the software. Image acquisition time was 1.5 s per volume. Patients were imaged between November of 2020 to October 2021.

Optical coherence tomography volumes were processed and presented in linear scale. Mean-intensity *en face* projections were calculated with custom software coded in MATLAB (18). B-scans presented in this manuscript were produced *via* image registration and averaging of adjacent B-scans. Three-dimensional image rendering was performed *via* the Volume Viewer plugin of Fiji, a distribution of ImageJ after pre-processing using a combination of thresholding and manual image segmentation (19). OCTA images were generated using a novel phase-stabilized complex-decorrelation methodology (20), with automated segmentation performed using a guided bidirectional graph search method (21), both of which were designed specifically for use in swept-source, widefield applications.

## Results

During the study period, we obtained images in 20 patients undergoing EUA in the operating room, as seen in Table 1. Here, we present a variety of pathologies and examples to illustrate some potential applications in pediatric retina.

**TABLE 1:** Patient ages and diagnoses.

	Diagnosis	Figure	Age
Case 1	Tractional retinal detachment (TRD) secondary to Familial Exudative Vitreoretinopathy (FEVR)	2A	3 years, 6 months
Case 2	TRD secondary to incontinentia pigmenti (IP)	2B, 8A	2B: 1 year, 6 months; 8A: 1 year
Case 3	TRD secondary to retinopathy of prematurity (ROP), Stage 4A	2C	4 months
Case 4	Rhegmatogenous retinal detachment (RRD)	3	5 years, 10 months
Case 5	Tractional and exudative retinal detachment (ERD) secondary to vasoproliferative lesion	4A	15 years
Case 6	Chronic exudative retinopathy	4B	16 years
Case 7	ERD secondary to ROP after laser	4C	5 months
Case 8	Coats disease	5	2 years, 3 months
Case 9	Retinoblastoma with calcified, partially calcified, and atrophic regressed tumors after completion of therapy	6A	3 years
Case 10	Retinoblastoma with partially calcified tumor in patient undergoing chemotherapy	6B	5 months
Case 11	Retinoblastoma with vitreous seeding and multifocal tumors	6C	7 months
Case 12	X-linked retinoschisis (XLRS)	7A	8 years
Case 13	Chorioretinal scarring with retinal traction secondary to non-accidental trauma (NAT)	7B	7 months
Case 14	ROP with regressed Stage 3 ROP with vitreoretinal traction	7C	2 months
Case 15	Persistent fetal vasculature (PFV)	7D	1 year, 7 months
Case 16	IP with peripheral avascular retina and neovascularization	8B	2 years, 7 months
Case 17	Hemangioblastomas in the setting of Von Hippel Lindau syndrome	9	15 years
Case 18	Central cataract in the setting of PFV	10A, 10B	6 months
Case 19	Retained silicone oil in the anterior chamber	10C	14 years
Case 20	TRD secondary to FEVR	11	4 months

*Patient ages are at the time of OCT imaging.*

### Retinal Detachments

**FIGURE 2:**
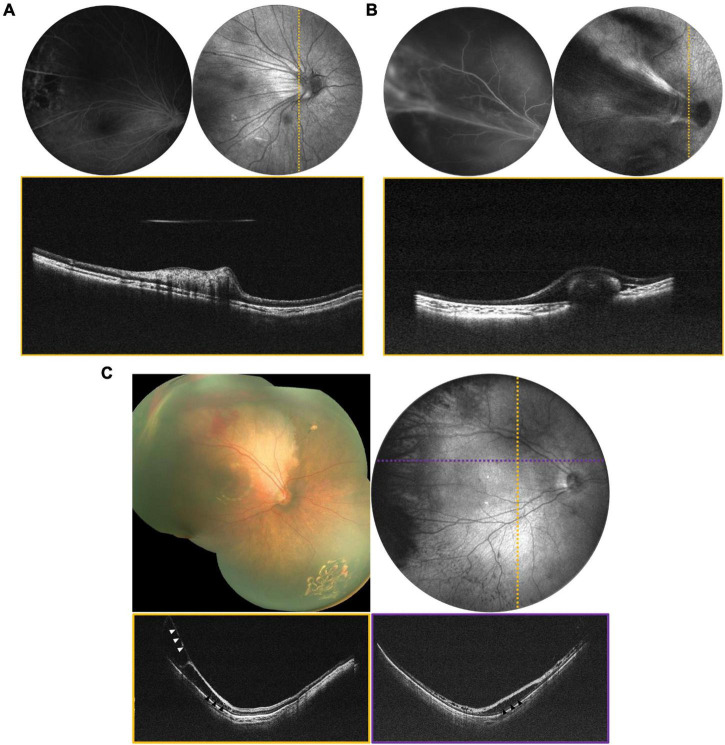
Tractional retinal disease evaluated *via* OCT. **(A)** Tractional retinal fold secondary to FEVR in a 3-year-old patient. **(B)** Tractional fold with peripheral detachment secondary to IP in a 1-year-old patient. For **(A,B)**, top left is the FA, top right is the mean-intensity *en face* projection taken with the 55° FOV configuration, and bottom is the B-scan corresponding to the dotted line. **(C)** Stage 4a ROP in a 4-month-old born at 24 weeks gestation. Top left image is a montage of fundus photographs. Top right is a 105° FOV OCT *en face* with dotted lines indicating the locations of the color-coded B-scans in the bottom row. Vitreoretinal traction is indicated by white arrows, and areas of schisis are indicated by black arrows.

**FIGURE 3:**
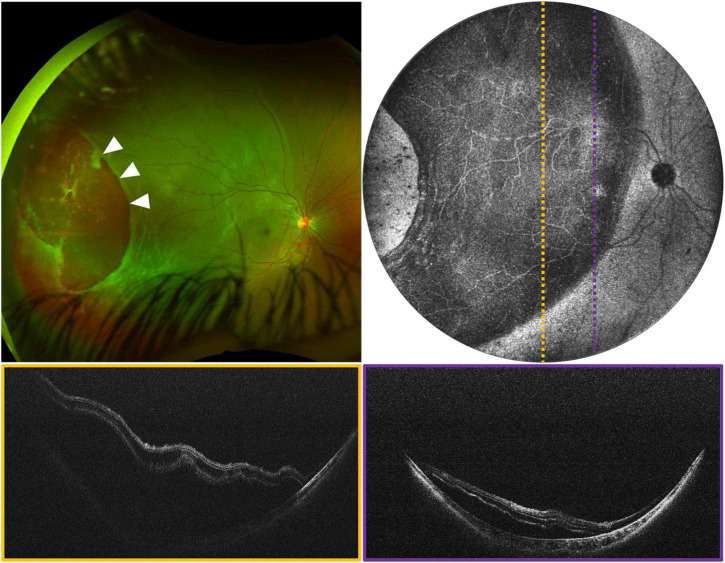
Rhegmatogenous retinal detachment (RRD) in a 5-year-old girl, evaluated using widefield OCT. **(Top left)** Image is an ultra-widefield fundus photograph, with white arrows indicating a large temporal break. **(Top right)** Image is the 105° FOV OCT *en face*, with dotted lines indicating the locations of the color-coded B-scans shown below.

**FIGURE 4:**
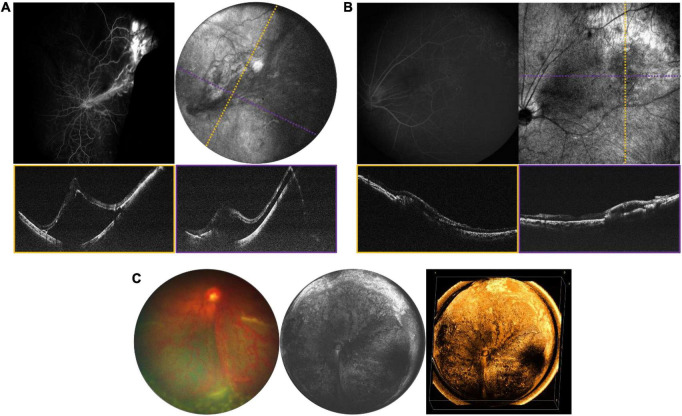
Tractional and exudative retinal detachments. **(A)** ERD in a 15-year-old girl in the setting of a vasoproliferative lesion. **(B)** Chronic exudative retinopathy in a 16-year-old girl. For **(A,B)**, top left image is fluorescein angiography (FA), top right is the mean-intensity *en face* projection [105° FOV for panel **(A)** and 40° FOV for panel **(B)**], with dotted lines indicating the locations of the color-coded B-scans shown below. **(C)** ERD in a 5-month-old patient with ROP. From left: fundus photograph, 105° FOV OCT en face, and three-dimensional rendering of the same OCT volume.

Portable widefield OCT facilitates the evaluation of tractional, exudative, rhegmatogenous (and combined mechanism) retinal detachments (RDs) in children. The most common visualization of OCT is the cross-sectional scan (B-scan) that reveals axial anatomy within a single imaging slice. However, SS-OCT can facilitate real-time *en-face* visualization of the entire imaging range. Figure 2 reveals *en face* and selected B-scans from several children with tractional retinal detachment (TRD). TRDs are most commonly related to peripheral epiretinal neovascularization with fibrosis, with the resulting vitreoretinal traction leading to macular dragging (as seen in Figure 2A), distortion of the normal retinal architecture, and if there is sufficient anterior-posterior traction, separation of the retina from the retinal pigment epithelium (RPE). OCT is more sensitive for detection of this spectrum of changes, as seen in Figure 2C which reveals an early stage 4a detachment in ROP with the selected B-scan demonstrating tractional schisis but no subretinal fluid. It is important to note that the transverse resolution of these B-scans is relatively low, the result of expanding FOV while maintaining efficient imaging time (1.5 s). Resolution can be improved with either longer imaging time, which is challenging in children, or narrower FOV, as previously seen with our 55° FOV prototype (11). Figure 3 demonstrates a rhegmatogenous retinal detachment (RRD) with a large temporal retinal break in a 5-year-old girl. Exudative retinal detachments (ERDs) may be relatively more common in pediatric retinal diseases such as in ROP after laser treatment or in severe Coats’ disease, which means they are often diagnosed clinically rather than with OCT imaging due to the limitations of existing commercially available devices. Yet accurate diagnosis is critical because the management of exudative detachments is often different than if the primary mechanism is tractional or rhegmatogenous. Figure 4 provides several examples ERDs and combined tractional and exudative RDs in children.

### Macular Exudation

**FIGURE 5:**
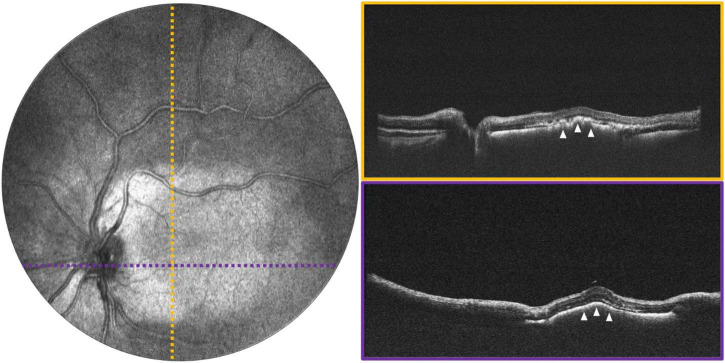
Macular exudation in a 2-year-old with Coats disease. **(Left)** Image shows 55° FOV OCT *en face* projection, with dotted lines indicating the locations of the color-coded B-scans shown on the **(right)**. White arrows point to the area of subretinal exudation.

While WF-OCT is critical for visualization of the retinal periphery, it can still be used to diagnose and monitor exudation in the macula in many diseases. Figure 5 demonstrates several examples of the visualization of subretinal exudative in Coats’ disease, including the potential benefit of *en face* visualization with topographic volume rendering. There are many previous publications focusing on the role of OCT in pediatric macular disease (2, 3, 22).

### Intraocular Tumors

**FIGURE 6:**
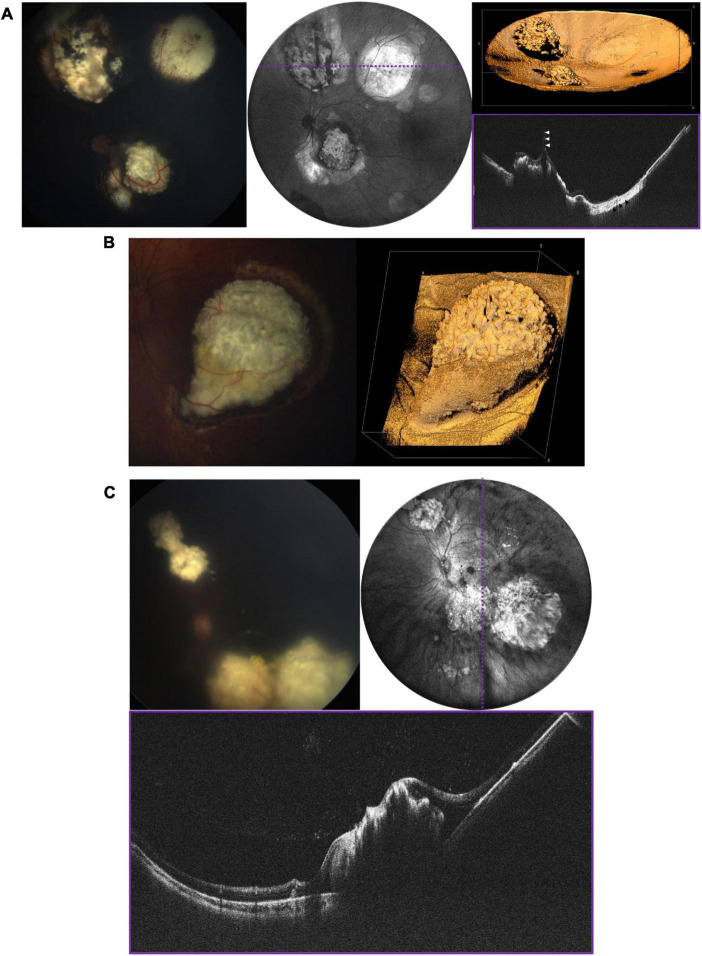
Retinoblastoma evaluated with OCT in three patients with bilateral disease. **(A)** 3-year-old with multifocal RB in the left eye who has completed therapy. Left image is the fundus photograph, middle image is the 105° FOV OCT *en face* projection showing multiple regressed tumors, with dashed purple line corresponding to the location of the B-scan on the bottom right. White arrows point toward a vitreous band, while black arrows point to an area of retinal atrophy. Top right image shows three-dimensional rendering of the same volume shown in the middle panel. **(B)** Large partially calcified retinoblastoma in a 5-month-old patient undergoing systemic chemotherapy. Fundus photograph is shown on the left, and three-dimensional rendering of 40° FOV, high-resolution OCT volume is shown on the right. **(C)** 7-month-old undergoing systemic chemotherapy with active RB including diffuse vitreous seeding and multifocal tumors in the left eye. Top left image shows the fundus photograph, top right shows the 105° FOV OCT *en face* projection, with dashed purple line corresponding to the B-scan below.

A number of retinal tumors can present in childhood including retinoblastoma (RB), retinal hemangioblastoma as part of Von-Hippel Lindau (VHL) disease, vasoproliferative tumor, and a variety of benign hamartomas such as choroidal hemangioma and congenital hypertrophy of the retina and RPE. The most serious of these is RB, which is both vision- and life-threatening. The current standard of care requires careful documentation of all tumors in the retina, including their size, location and the presence of any associated vitreous seeding, and subretinal fluid. Fundus photos are used to document these findings. Commercially available OCT systems are also widely used in retinoblastoma care, but have significant limitations due to their narrow field of view and narrow depth of focus (23–26). Retinoblastoma tumors are often highly elevated, multifocal and arise in the peripheral retina as well as the posterior pole. There is considerable potential for the use of WF-OCT in retinoblastoma care. Figure 6 demonstrates several examples of RB documented with our device during routine RB EUAs. The WF-OCT system was successful in capturing three dimensional images of elevated tumors, including those in the far periphery, and provided better images than traditional fundus photography in the setting of diffuse vitreous seeding (Figure 6C). WF-OCT was also able to identify very small subclinical tumors (27), indicating that it may prove useful in surveillance for new tumors in children with known RB or in those being screened due to family history of the disease.

### Vitreoretinal Interface Disorders

**FIGURE 7:**
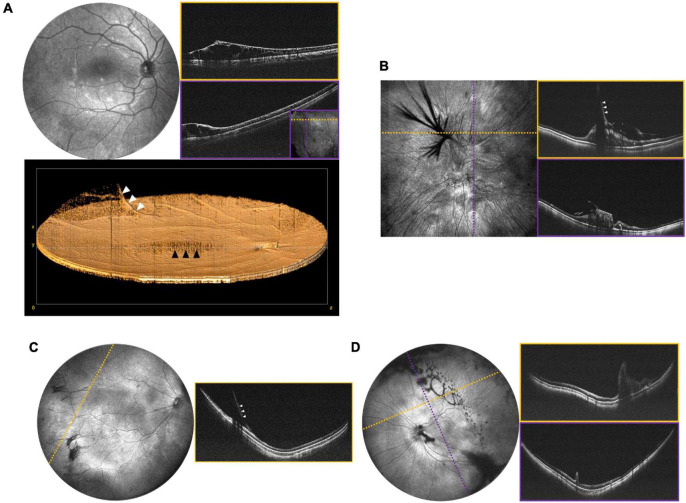
Peripheral retinal and vitreoretinal interface abnormalities. **(A)** OCT evaluation in an 8-year-old patient with XLRS. Top left shows the 55° FOV *en face* projection, with corresponding volume rendering shown below. White arrows denote a blood vessel extending into the area of vitreoschisis (vitreous veils) and black arrows denote the area of foveoschisis. Top right images show high-resolution scans of an area of retinoschisis taken with 40° FOV. The inset in the bottom-right corner of the purple B-scan shows the locations of the cross-sections in dotted lines. **(B)** OCT evaluation of 7-month-old patient with history of non-accidental trauma, displaying disorganization of the retinal architecture, as well as vitreoretinal traction in region of prior breakthrough vitreous hemorrhage. Left image shows a 40° FOV *en face* projection, with dashed yellow and purple lines denoting the location of the corresponding B-scans on the right. White arrows denote vitreoretinal traction. **(C)** Vitreoretinal traction in a 2-month-old at site of regressed stage 3 extraretinal neovascularization in ROP. Left image shows 105° FOV *en face* view with dashed line indicating the location of the cross-sectional B-scan pictured on the right. White arrows denote vitreoretinal traction. **(D)** 1-month-old with ectopic PFV. Top left image shows the 105° FOV *en face* projection with complex oval-shaped vitreoschisis. Dotted lines correspond to B-scan locations demonstrating retinal fold through the macula.

Changes at the vitreoretinal interface are better diagnosed with OCT than ophthalmoscopy and are common in pediatric proliferative retinopathies, some inherited retinal degenerations, and disorders of ocular development. Figure 7A shows an example of X-linked retinoschisis (XLRS), in which retinoschisis may manifest both in the macula and periphery. Many conditions demonstrate an abnormally adherent vitreoretinal interface. An abnormal vitreoretinal interface may be associated with prior trauma, as in Figure 7B, and regressed neovascularization in ROP, as in Figure 7C. Finally, in persistent fetal vasculature (PFV), there is a cellular connection through the vitreous cavity that connects the retina to the anterior segment, which can be associated with traction at the nerve or in an extramacular location, as seen in Figure 7D.

### Vascular Disorders

**FIGURE 8:**
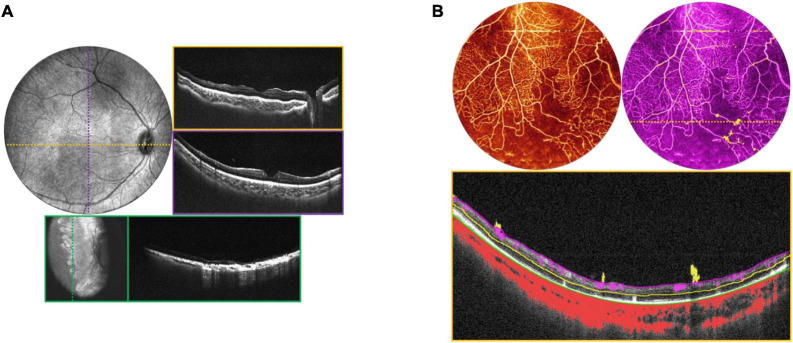
Incontinentia pigmenti (IP). **(A)** OCT volume of a 1-year-old with IP. Top left image is the OCT 55° FOV *en face* projection. Corresponding color-coded B-scans at top right show irregularities in the nerve fiber layer and retinal surface. Bottom image in teal shows the retina after laser treatment, with *en face* view on the left, and corresponding B-scan on the right. **(B)** OCTA evaluation of a 2-year-old patient with IP. Top left image shows the OCTA en face projection with 55° FOV, while top right image shows the same volume with extraretinal neovascularization highlighted in bright yellow. Bottom image is the B-scan corresponding to the dashed yellow line above, showing automated segmentation of capillary plexus layers.

**FIGURE 9:**
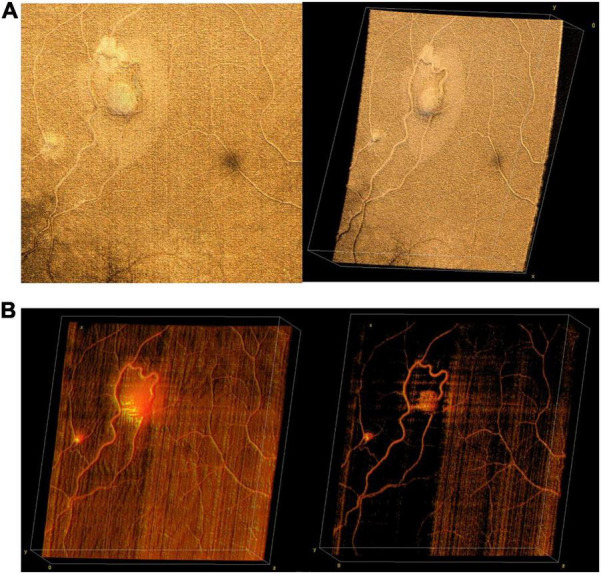
Retinal hemangioblastomas in the setting of Von Hippel Lindau (VHL) syndrome. **(A)** Volume rendering of retinal hemangioblastomas in a 15-year-old. Left image shows an *en face* of a three-dimensional volume rendering of the tumors taken with a 40° FOV, while image on the right shows an angled view of the same volume. **(B)** Three-dimensional visualization of vessels generated from OCTA of the same volume as in panel **(A)**. The image on the left shows a three-dimensional rendering of the OCTA volume, showing flow signal within the tumors. Image on the right shows the same volume with higher contrast between vessels and surrounding tissue.

Obtaining high quality OCTA is challenging even in cooperative adults, more so in children, and even more so with wider field of view. Nontheless, particularly under anesthesia, it is possible to explore the potential role OCTA may play in the diagnosis of pediatric retinal diseases when used in conjunction with structural OCT (Figure 8A). Figure 8B demonstrates an OCTA taken during an EUA for a child with incontinentia pigmenti (IP), revealing both non-perfusion and neovascularization without the need for fluorescein dye. Figure 9 demonstrates several VHL tumors visualized with *en face* OCT and OCTA.

### Anterior Segment Optical Coherence Tomography

**FIGURE 10:**
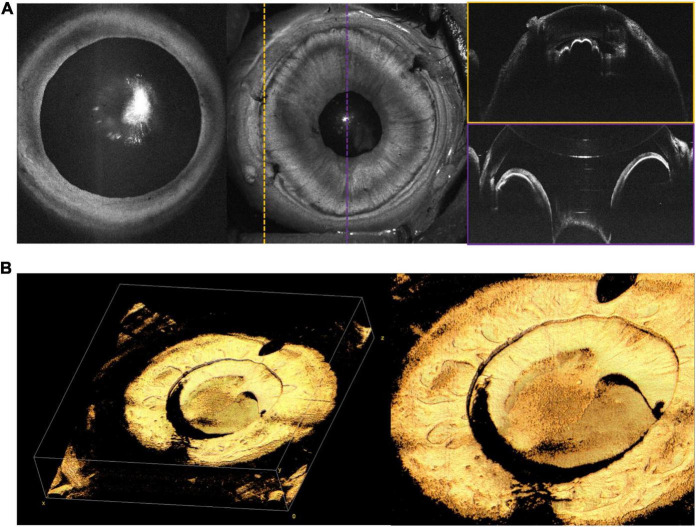
Anterior segment (AS)-OCT. **(A)** AS-OCT in a 6-month-old with PFV. Leftmost *en face* OCT demonstrates good pupillary dilation and central cataract. Middle image shows *en face* of AS-OCT, with corresponding color-coded B-scans on the right. The scan outlined in yellow was taken at the limbus, providing visualization of the ciliary body. The scan outlined in purple shows the iris, with a reflection artifact affecting the cornea. **(B)** Silicone oil in the anterior chamber on AS-OCT in a 14-year-old. Leftmost image shows three-dimensional rendering of the iris and anterior chamber, while rightmost image shows a close up of retained oil above the lens. Both **(A,B)** were captured with a 105° FOV.

Anterior segment (AS)-OCT has demonstrated a number of potential uses in adults, including evaluation of corneal curvature and pathology, angle structures, and iris and lens abnormalities (28). We have included a few examples of AS-OCT obtained in our practice, but believe that the most significant potential application of this imaging may be in the evaluation and management of pediatric glaucoma in which anterior segment dysgenesis is typical (29). Figure 10 reveals *en face* and cross-sectional AS-OCT in several patients with both preoperative and post-operative abnormalities of the anterior segment.

## Discussion

In this paper, we reviewed our experience using WF-OCT in the management of patients with a variety of pediatric retinal diseases undergoing EUA. Compared to the highest resolution commercially available adult OCT devices, our prototype uses a faster, swept-source laser, which facilitates efficient imaging of the retina even in awake neonates and children. These results demonstrate the tradeoff between FOV and resolution, which is necessary when trying to keep imaging time to a minimum.

### Retinopathy of Prematurity

The diagnosis of ROP relies on subjective assessment of clinical features on ophthalmoscopic exam or fundus photography, despite significant inter-observer variability in diagnosis, practice, and outcomes. Most of the early work using OCT work has focused on macular manifestations of ROP, such as the presence of macular edema, vitreous opacities, and the presence of retinoschisis posterior to the ridge (3, 4, 30). Widefield OCT has demonstrated the potential to provide real-time *en face* visualization, objective assessment of the peripheral stage, longitudinal monitoring of disease progression and regression, and detection of early vitreoretinal interface abnormalities (13, 14, 31).

### Tractional, Exudative, and Rhegmatogenous Retinal Detachments

**FIGURE 11:**
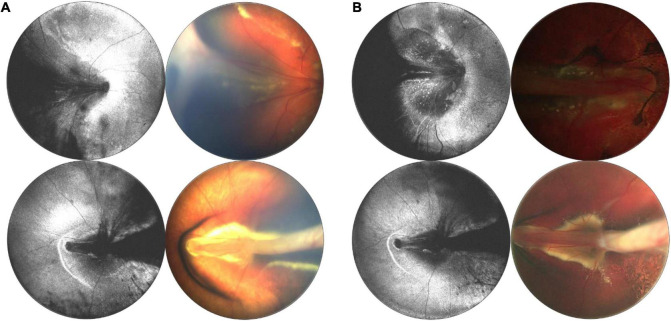
Longitudinal monitoring of a patient with familial exudative vitreoretinopathy (FEVR). **(A)** Pre-surgical repair of bilateral tractional retinal detachments secondary to FEVR in a 4-month-old patient taken with 55° FOV device. **(B)** Post-surgical repair images, with OCT *en face* taken 1 week after surgery, and fundus photographs taken 3 months after surgery. For panels **(A,B)**, left column are OCT en face projections, whilst right column are color fundus photographs.

Differentiating the cause of retinal detachment is key for proper management of retinal detachments in children, and OCT may be a pivotal tool. RRD repair depends on accurate identification of breaks, and the identification and management of proliferative vitreoretinopathy (PVR) membranes, which may be above or below the retinal surface. The standard of care is to carefully observe the entire retina with ophthalmoscopy and scleral depression for the presence of breaks. However, clinical diagnosis is not perfect and OCT may be superior for identification of peripheral pathology (32, 33). In this paper, we have presented several examples of tractional, rhegmatogenous, and exudative detachments and highlighted ways in which WF-OCT may be utilized in the diagnosis and monitoring of these diseases in the future. One example of a potential use of WF-OCT is in monitoring the resolution of subretinal fluid following RD surgery. Figure 11 reveals pre- and post-operative *en face* OCT and B-scans for a child with familial exudative vitreoretinopathy (FEVR) who presented shortly after birth with bilateral retinal folds and tractional-exudative RDs. Post-operative scans reveal improved exudation and subretinal fluid.

### Intraocular Tumors

In retinoblastoma (RB), the most common primary intraocular malignancy in children, the value of OCT has been demonstrated, however, there are known challenges with commercially available OCT systems (23–26). The potential value for WF-OCT to image and document tumor location and size, to monitor treatment response to laser, cryotherapy and chemotherapy, and to evaluate for newly emerging tumors is clear. A system which could combine structural images with OCTA would be of particular interest. House et al. (34) utilized OCTA to evaluate irregular tumor vasculature, with the advantage of depth resolution compared to fluorescein angiography (FA). As tumor vascular density in RB has been found to correlate significantly with a greater risk of metastasis (35), OCTA imaging could be useful in providing prognostic information. Beyond RB, there is considerable potential for WF-OCTA in the management of a wide variety of elevated and/or peripheral tumors involving the choroid and retina. In retinal hemangioblastomas, which may be associated with Von Hippel Lindau (VHL) syndrome, OCTA has been useful to differentiate non-vascular lesions from the vascular tumors, but the limited FOV has been a comparative disadvantage versus FA (36).

### Inherited Retinal Dystrophies and Congenital Anomalies

In the realm of inherited retinal dystrophies (IRDs), OCT has been useful in identifying prognostic indicators, such as foveal cavitation (37), and the extent of photoreceptor atrophy. Spectral-domain (SD-OCT) technology has provided adequate axial resolution to evaluate X-linked retinoschisis in greater detail, elucidating the precise layers where retinal separation tends to occur (38). OCTA has also been utilized to evaluate choroidal neovascularization in IRDs, with advances in automated image segmentation capable of accurately delineating vascular plexuses even in the setting of distorted retinal architecture (39). In FEVR, OCTA has shown vascular abnormalities in the deep and superficial vascular complexes (40), and for both FEVR and PFV, handheld OCT has been used to detect optic nerve head dragging and associated vitreous bands (41, 42). In IP, OCT has illustrated subclinical change to foveal structure, including inner and outer retinal thinning associated with retinal ischemia in IP (43–46).

### Limitations of Widefield Optical Coherence Tomography Imaging

As mentioned throughout, there is a tradeoff between FOV, resolution, and acquisition speed, therefore the transverse resolution is lower for a given laser when expanding the FOV for a given amount of imaging time. Practically speaking, that means that individual B-scans may be lower resolution using this approach compared to commercially available systems with narrower FOV, such as the Heidelberg Flex system, although the acquisition time is faster (6). Other limitations to this approach overlap with those found in comparable commercial OCT systems, and include motion artifacts and shadow which often necessitate the capture of multiple redundant volumes per region of interest. Current OCT systems are also limited by the potential axial imaging range, which limits the ability to obtain UWF imaging in larger eyes.

## Conclusion

In summary, the use of WF-OCT has several potential advantages compared to the clinical exam and fundus photography in the setting of pediatric retinal diseases. As in adults, the axial resolution is superior to what our eyes can see, enabling earlier detection of retinal abnormalities in multiple diseases. *En face* visualization can provide the same benefit as fundus photography, but with volumetric structural and angiographic information as well. Finally, OCT facilitates objective assessment of retinal structures that can be used to monitor disease stability. The challenges to widespread adoption of this technology remain the lack of commercially available OCT devices of sufficient speed and FOV to be effective in capturing images outside of the macula. As the costs of lasers come down with time, our hope is that the market will facilitate the routine use of this technology in the care of children with retinal disease.

## Data Availability Statement

The original contributions presented in this study are included in the article/supplementary material, further inquiries can be directed to the corresponding author.

## Ethics Statement

The studies involving human participants were reviewed and approved by the Oregon Health and Science University Institutional Review Board. Written informed consent was obtained from the minor(s)’ legal guardian/next of kin for the publication of any potentially identifiable images or data included in this article.

## Author Contributions

JC, MC, DH, YaJ, and YiJ designed the study and obtained the funding. SO developed and maintained the patient database and consented all patients. AS contributed to the data and critically reviewed the manuscript. T-TN, GL, SN, SK, and XW identified, processed, and contributed to the images to the final manuscript. All authors reviewed and approved of the final version of the manuscript.

## Conflict of Interest

Oregon Health and Science University (OHSU), DH, and YaJ have significant financial interests in Optovue, a company that may have a commercial interest in the results of this research and technology. These potential conflicts of interest have been reviewed and managed by OHSU. DH and YaJ have received royalties for patent files through OHSU, as well as loaned equipment for research from Optovue. The remaining authors declare that the research was conducted in the absence of any commercial or financial relationships that could be construed as a potential conflict of interest.

## Publisher’s Note

All claims expressed in this article are solely those of the authors and do not necessarily represent those of their affiliated organizations, or those of the publisher, the editors and the reviewers. Any product that may be evaluated in this article, or claim that may be made by its manufacturer, is not guaranteed or endorsed by the publisher.
